# Prognostic Modeling for Bone Sarcomas Based on a Large Prospective Cohort From a Tertiary Care Cancer Center in India

**DOI:** 10.1200/GO.24.00142

**Published:** 2025-02-06

**Authors:** Jyoti Bajpai, Laboni Sarkar, Sushmita Rath, Akash Pawar, Arun Chandrashekharan, Goutam Panda, Dharmpal Jakar, Jaya Ghosh, Siddhartha Laskar, Bharat Rekhi, Nehal Khanna, Jifmi Jose, Mukta Ramdawar, Nilendu Purandare, Prabhat Bhargava, Nivedita Chakrabarty, Kunal Gala, Yogesh Kembhavi, Venkatesh Rangarajan, Shripad Banavali, Sudeep Gupta

**Affiliations:** ^1^ Department of Medical Oncology, Tata Memorial Centre, Homi Bhabha National Institute (HBNI), Mumbai, India; ^2^ Department of Biostatistics, Tata Memorial Centre, Homi Bhabha National Institute (HBNI), Mumbai, India; ^3^ Department of Radiation Oncology, Tata Memorial Centre, Homi Bhabha National Institute (HBNI), Mumbai, India; ^4^ Department of Pathology, Tata Memorial Centre, Homi Bhabha National Institute (HBNI), Mumbai, India; ^5^ Department of Nuclear Medicine, Tata Memorial Centre, Homi Bhabha National Institute (HBNI), Mumbai, India; ^6^ Department of Radiodiagnosis, Tata Memorial Centre, Homi Bhabha National Institute (HBNI), Mumbai, India

## Abstract

**PURPOSE:**

Outcomes of adolescents and young adults (AYA) with bone sarcomas including osteosarcoma (OGS) and Ewing sarcoma (ES) are affected by various factors including inadvertent previous treatment and poor compliance. We aimed to develop a risk-scoring system derived and validated at a tertiary care cancer center in India.

**METHODS:**

All AYA OGS and ES cases treated at our institute with OGS-12 and Ewing’s family of tumors-2001 (EFT-2001) protocols from 2011 to 2021 and 2013 to 2018, respectively, were prospectively analyzed. Weighted scores provided to each prognostic variable on the basis of approximate ratios of the beta coefficients of each factor in the multivariable model were summated to divide patients into three clinically discriminatory risk groups, validated by applying separately to derivation, validation, and whole cohorts.

**RESULTS:**

Among 606 (81.0%) of 748 AYA with nonmetastatic OGS, significant factors included in the prognostic model were failure to complete protocol (hazard ratio [HR], 2.65), previous treatment (HR, 2.93), necrosis <90% (HR, 1.63), joint involvement (HR, 2.0), and serum alkaline phosphatase >median (204 U/L; HR, 1.63). Of 104 (39.5%) of 263 AYA with ES, significant factors were failure to complete protocol (HR, 2.84), previous treatment (HR, 6.37), necrosis <100% (HR, 8.73), and tumor size >8 cm (HR, 2.64). For 142 (38.8%) of 366 AYA with metastatic OGS, significant factors were failure to complete protocol (HR, 5.29), metastases not amenable to local treatment (HR, 1.96), necrosis <90% (HR, 1.96), and >10 metastases (HR, 2.44). For 38 (43.6%) of 82 AYA with metastatic extremity ES, significant factors were failure to complete protocol (HR, 3.88) and metastases not amenable to local treatment (HR, 10.6).

**CONCLUSION:**

We developed simple, effective prognostic models for AYA with bone sarcomas with specific potential relevance for low- and middle-income countries.

## INTRODUCTION

Bone sarcomas in adolescents and young adults (AYA) inclusive of osteosarcoma (OGS) and Ewing sarcoma (ES) are rare, aggressive yet chemotherapy-sensitive tumors with variable survival rates in low- and middle-income countries (LMIC). There are various factors including delayed presentation with advanced disease and higher rates of metastatic disease, inadvertent previous treatment by primary care physicians, high dropout rates, and poor adherence due to socioeconomic constraints with resultant compromised outcomes. Non–high-dose methotrexate (Non-HDMTX)–based regimens for OGS have gained popularity in these regions due to ease of administration on daycare basis, low cost, and convenience. Therefore, finding valuable prognostic factors specifically targeting AYA in LMIC is crucial for predicting high-risk patients and early intervention to improve survival rates.

**CONTEXT**

**Key Objective**
Outcomes of adolescents and young adults (AYA) with bone sarcomas including osteosarcoma (OGS) and Ewing sarcoma (ES) are affected by various underexplored factors including inadvertent previous treatment and poor compliance; with this in mind, we aimed to develop risk-scoring systems for AYA with bone sarcomas at a tertiary care center in India.
**Knowledge Generated**
In addition to conventional prognosticators such as tumor size, elevated alkaline phosphatase, joint involvement, and histological necrosis, failure to complete the treatment protocol (all cohorts) and the disease not being amenable to curative treatment (metastatic bone sarcomas) were associated with inferior outcomes. Prognostic models were developed separately for nonmetastatic and metastatic OGS and ES on the basis of weighted scores provided to each prognostic variable.
**Relevance**
The simple prognostic models developed and validated at our institute are effective and with potential wide applicability in low- and middle-income countries and merit appropriate recognition.

Among patients with OGS, possible prognostic factors, tumor size, metastatic disease at the time of diagnosis, histological grade, histologic response to neoadjuvant chemotherapy (NACT), and adequate surgical margins have consistently shown a strong correlation with survival.^1,2^ Nonuniformity of chemotherapy protocols for OGS precludes generalizability of results. We have previously published our experience with our institutional standard low-cost, non-HDMTX–based OGS-12 protocol where serum alkaline phosphatase (SAP) level for event-free survival (EFS) and performance status for overall survival (OS) were found to be independent prognosticators, concordant with other reports, including those from India.^3-6^ Histological response to NACT was an independent predictor of both EFS and OS, which is also well established.

For ES, multiple prognostic factors have been reported, such as age, gender, localization, volume and size of the primary tumor, presence of metastasis, treatment regimens, a baseline level of hemoglobin or lactate dehydrogenase (LDH), and pathologic response to NACT.^7-9^ In our published data on patients with ES at our institute treated with Ewing’s family of tumors-2001 (EFT-2001) protocol, statistically significant prognostic factors included longer symptom duration, ≥99% necrosis, and protocol completion.^10^

There are unique challenges with rare cancers like bone sarcomas globally and a prognostic model with wide applicability including for patients in LMIC wherein including social challenges also play a role is an unmet need. Special challenges in LMIC include lack of awareness among patients and primary health care providers with resultant delayed presentation, upstaging, and high tumor burden.

We aimed to identify prognostic factors including those specific to LMIC. Additionally, we derived and validated a prognostic score for AYA patients with OGS and ES on the basis of our cohort that integrates biologic and social factors, with potential applications for LMIC settings.

## METHODS

All AYA OGS and ES cases treated at Tata Memorial Center, Mumbai, with OGS-12 and EFT-2001 protocols from November 2011 to January 2021, and January 2013-November 2018, respectively, were prospectively analyzed separately. The OGS-12 protocol consists of doublets of doxorubicin, ifosfamide, and cisplatin given sequentially for eight cycles in both NACT and adjuvant chemotherapy (ACT) settings. The EFT-2001 protocol consists of a 12-month course of ifosfamide plus etoposide and vincristine, doxorubicin plus cyclophosphamide. Demographic factors recorded were age, gender, and nutritional parameters. Disease factors were symptom duration, LDH, SAP, tumor size, presence and sites of metastases, pathological fracture, and neurovascular bundle involvement. Patients were stratified on the basis of age into categories 15-25 and >25-39 to evaluate the prognostic significance of age. Patients with metastatic disease were stratified into categories on the basis of number (≤10, >10), location (lung, bone, or other), and whether metastases were amenable to local treatment on the basis of multimodality joint clinic discussion. Toxicities were documented using the US National Cancer Institute-Common Toxicity Criteria for Adverse Events version 4.0. Histological response on the surgical specimen was assessed using Huvo's necrosis grading, wherein good responders were defined as those with <10% viable cells.^11^

Treatment characteristics recorded from electronic medical records included markers of noncompliance (failure to complete protocol and failure to complete treatment within the planned period with 25% additional time to allow for justifiable reasons for noncompliance), previous inadvertent treatment by peripheral practitioners, and febrile neutropenia as a marker of chemosensitivity.

The study was conducted in accordance with ethical principles outlined in the Declaration of Helsinki (Fortaleza, Brazil 2013), International Council for Harmonization-Good Clinical Practice, European Directive 2001/20/EC, US Code of Federal Regulations, South African Good Clinical Practice Guidelines, and other institutional regulatory requirements. Data collection and analyses were conducted in a single institution. This study is registered with Clinical Trials Registry—India (CTRI) identifier: CTRI/2023/10/059247.

### Statistical Analysis

Data were analyzed using IBM SPSS version 25 Inc, Chicago, IL and RStudio software version 2023.03.0. Descriptive statistics were represented as median or percentages. T tests or linear regression models were used to analyze continuous measures, and chi-square tests, tests of proportions, and logistic regressions were used to analyze binary data. Survival was estimated using the Kaplan-Meier method and compared using a log-rank test. The primary outcome was EFS. Secondary outcomes were OS and histological necrosis. The effect of covariates on survival was estimated using the Cox proportional hazards analysis. Significant factors on univariable analysis were tested in multivariable analysis.

### Generation of the Derivation and Validation Cohorts and Identification of Prognostic Factors in the Derivation Cohort

The whole cohort was divided in a 2:1 ratio into a derivation and validation cohort in a randomized fashion. Univariable Cox regression analyses identified factors prognostic for EFS in the derivation cohort. Factors with *P* < .05 on univariable analyses were included for multivariable analysis in a backward stepwise fashion on the basis of likelihood ratio. Factors with *P* < .05 in the final multivariable model in the derivation cohort were used to formulate the risk score.

### Formulation of Risk Score

A weighted score was provided to each prognostic variable. The score was computed on the basis of the approximate ratios of the beta coefficients of each factor in the multivariable model. The total score was calculated by summation of individual prognostic factor scores and was used to divide patients into three clinically discriminatory risk groups.

### Validation of the Risk Score

The risk score was validated by applying it separately to the derivation, validation, and whole cohorts. Kaplan-Meier curves were constructed to represent EFS and OS in the three risk groups in each of the three cohorts. Harrell's concordance index (c-index) was calculated for estimating the predictive ability of the risk category model for EFS and OS in the derivation, validation, and whole cohorts. A receiver operating characteristic (ROC) curve was also constructed by comparing the predicted and actual 18-month and 36-month EFS and OS in each of the three cohorts, and the timed area under the ROC curve for the derivation, validation, and whole cohorts was estimated.

## RESULTS

### Baseline Characteristics

**FIG 1:**
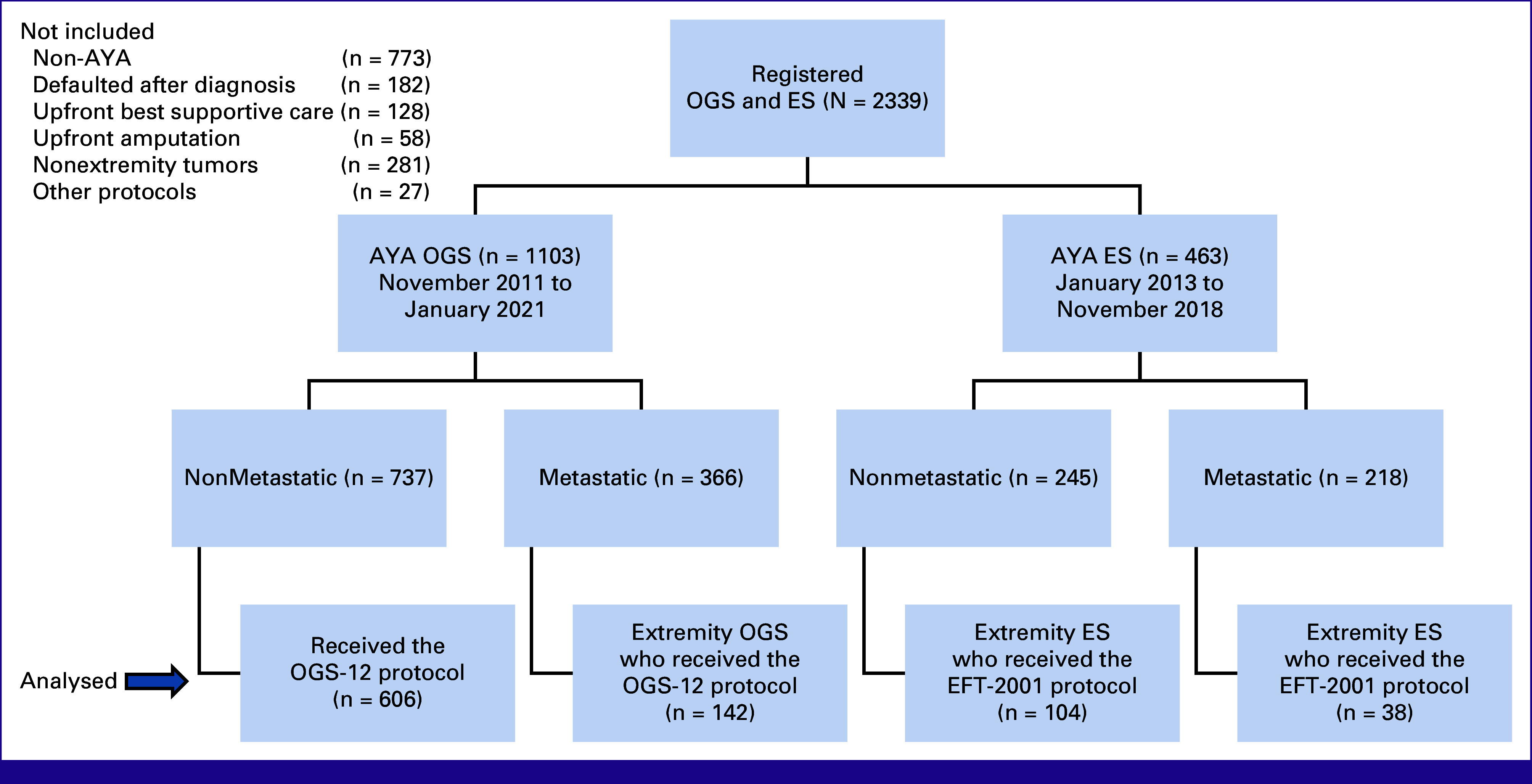
CONSORT diagram depicting the number of patients analyzed in the current study. AYA, adolescents and young adults; ES, Ewing sarcoma; OGS, osteosarcoma.

Of the 2,399 patients with bone sarcomas registered during the respective study period, 1,566 were AYA patients (age 15-39 years; Fig 1). This included 1,103 AYA with OGS registered during the study period November 2011-January 2021, of whom 748 were AYA patients with extremity OGSs uniformly treated with the OGS-12 protocol as first-line therapy, consisting of NACT, surgery, and ACT, comprising 606 (81.0%) with nonmetastatic disease and 142 (19.0%) with metastatic tumors included in the final analysis. There were 142 AYA with ES of extremities treated with the EFT-2001 protocol during the study period January 2013-November 2018, comprising 104 (73.2%) with nonmetastatic tumors and 38 (26.8%) with metastatic disease included in the final analysis. Baseline characteristics are detailed in Tables 1 and 2.

**TABLE 1:** Baseline and Treatment Characteristics: Osteosarcoma

Demographics	Nonmetastatic (n = 606)	Metastatic (n = 142)
Age, years, median (IQR)	19 (15-39)	18 (15-39)
Sex, No. (%)		
Males	446 (74)	104 (73)
Females	160 (26)	38 (27)
Primary site, No. (%)		
Femur	317 (52)	71 (50)
Tibia	191 (32)	49 (34)
Humerus	53 (9)	17 (12)
Fibula	19 (3)	1 (1)
Other	26 (4)	4 (3)
Histology		
Conventional	508 (84)	124 (85)
Chondroblastic	62 (10)	14 (10)
Telangiectatic	22 (4)	5 (3)
Fibroblastic	12 (2)	2 (2)
Periosteal	2 (<1)	**—**
Nutritional parameters, No. (%)	Nonmetastatic (n = 561)	Metastatic (n = 127)
Deficient albumin (<3.5 g/dL)	28 (5)	9 (7)
Anemia	181 (32)	48 (38)
Iron deficiency	295 (52)	37 (29)
B12 deficiency	158 (28)	44 (35)
Folate deficiency	24 (4)	3 (2)
Tumor characteristics	Nonmetastatic (n = 561)	Metastatic (n = 127)
Symptom duration, months, median (IQR)	3 (0.25-24)	3 (1-18)
Elevated lactate dehydrogenase (>190 U/L), No. (%)	261 (47)	70 (49)
Elevated serum alkaline phosphatase (>120 U/L), No. (%)	454 (81)	113 (80)
Neurovascular involvement, No. (%)	83 (15)	20 (16)
Pathological fracture, No. (%)	38 (7)	12 (9.4)
Joint involvement, No. (%)	185 (33)	47 (37)
Tumor size, cm, median (IQR)	9.75 (3-36)	11.65 (5-30)
Treatment characteristics	Nonmetastatic (n = 606)	Metastatic (n = 142)
Previous treatment, No. (%)	45 (7)	15 (10)
Treatment characteristics: ITT cohort	Nonmetastatic (n = 561)	Metastatic (n = 127)
Protocol completion, No. (%)	481 (86)	83 (65)
Treatment characteristics: per-protocol cohort	Nonmetastatic (n = 481)	Metastatic (n = 83)
Treatment duration, median (IQR)	26 (21-47) weeks	29 (23-51) months
Compliance, No. (%)	416 (86)	55 (66)
Histological necrosis (Huvo's grading), No. (%)	Nonmetastatic (n = 495)	Metastatic (n = 111)
≥90%	298 (60)	55 (50)
100%	91 (18)	20 (18)
Toxicity, No. (%)	Nonmetastatic (n = 606)	Metastatic (n = 142)
Febrile neutropenia	264 (44)	53 (37)
Dose modifications	121 (20)	48 (34)

Abbreviation: ITT, intention-to-treat.

**TABLE 2:** Baseline and Demographic Characteristics (Ewing's sarcoma)

Baseline Characteristics	Nonmetastatic (n = 104)	Metastatic (n = 38)
Demographics		
Age, years, median (IQR)	21 (15-39)	21 (15-35)
Sex, No. (%)		
Males	69 (66)	27 (71)
Females	35 (34)	11 (29)
Nutritional parameters, No. (%)	Nonmetastatic (n = 91)	Metastatic (n = 35)
Deficient albumin (<3.5 g/dL)	3 (3.3)	2 (5.7)
Anemia	34 (37)	22 (63)
Disease characteristics	Nonmetastatic (n = 91)	Metastatic (n = 35)
Symptom duration, months, median (IQR)	6 (1-36)	4 (1-12)
Elevated lactate dehydrogenase (>190 U/L), No. (%)	13 (14)	9 (26)
Tumor size, cm, median (IQR)	8.8 (1.6-22)	9.5 (2-22)
Treatment characteristics, No. (%)	Nonmetastatic (n = 104)	Metastatic (n = 38)
Previous treatment	13 (13)	3 (8)
Treatment characteristics: ITT cohort, No. (%)	Nonmetastatic (n = 91)	Metastatic (n = 35)
Protocol completion	70 (77)	20 (57)
Treatment characteristics: per-protocol cohort	Nonmetastatic (n =70)	Metastatic (n = 20)
Treatment duration, weeks, median (IQR)	48 (27-111)	49 (27-91)
Compliance, No. (%)	51 (73)	13 (65)
Histological necrosis, No. (%)	Nonmetastatic (n =72)	Metastatic (n = 22)
≥90%	32 (44)	10 (45)
100%	22 (30)	5 (23)
Toxicity, No. (%)	Nonmetastatic (n = 104)	Metastatic (n = 38)
Febrile neutropenia	54 (52)	16 (42)
Dose modifications	24 (23)	14 (37)

Abbreviation: ITT, intention-to-treat.

### Treatment Characteristics

Histological necrosis and chemotoxicity: These are detailed in Tables 1 and 2.

### Treatment Compliance

Of the 1,566 registered AYA with bone sarcomas, 182 (11.6%) defaulted after diagnosis. Among the treatment-naïve cohorts enrolled, respectively, on the OGS-12 and EFT-2001 protocols, 42 (6.1%) with OGS and 18 (14.2%) with ES abandoned the treatment subsequently (Tables 1 and 2).

### Outcomes

For nonmetastatic OGS, at a median follow-up of 59.7 (95% CI, 54.4 to 63.3) months, the 5-year EFS (5-EFS) was 58.6% (95% CI, 54.1 to 63.5) and the 5-year OS (5-OS) was 75% (95% CI, 70.9 to 79.5). For metastatic OGS, at a median follow-up of 47.3 (95% CI, 36.2 to 68.2) months, the 5-year EFS was 18.7% (95% CI, 12.7 to 27.5) and the 5-year OS was 36.7% (95% CI, 28.2 to 47.8). For nonmetastatic ES, at a median follow-up of 72.1 (95% CI, 66.6 to 83.5) months, the 5-year EFS was 66.4% (95% CI, 57.6 to 76.7) and the 5-year OS was 80.4% (95% CI, 72.6 to 89.0). For metastatic ES, at a median follow-up of 70.5 (95% CI, 38.7 to not reached) months, the 5-year EFS was 21.9% (95% CI, 11.4 to 42.0 and the 5-year OS was 43.7% (95% CI, 28.5 to 67).

### Prognostic Factors

#### OGS

For nonmetastatic OGS, factors found significant on univariable analysis are shown in Table 3. On multivariable analysis, higher baseline SAP, neurovascular bundle involvement, joint involvement, poor histological necrosis, failure to complete treatment, and previous treatment independently predicted inferior EFS.

**TABLE 3:** Nonmetastatic Osteosarcoma: Factors Significant on Univariable and Multivariable Analyses

Parameters	Univariable		Multivariable	
	HR (95% CI)	*P*	HR (95% CI)	*P*
SAP >median (204 U/L)	1.69 (1.3 to 2.1)	.0001	1.63 (1.24 to 2.14)	.0001
LDH >median (224 U/L)	**1.70 (1.3** to **2.1)**	**.0001**	1.22 (0.6 to 2.4)	.578
Tumor size >12 cm	**1.07 (1.0** to **1.1)**	**.046**	1.05 (0.72 to 1.53)	.776
NVB involvement	**2.5 (1.8** to **3.3)**	**.0001**	**1.67 (1.18** to **2.37)**	**.004**
Joint involvement	**2.29 (1.7** to **2.9)**	**.0001**	**2.0 (1.49** to **2.69)**	**.0001**
Failure to complete protocol	**3.6 (2.6** to **5.0)**	**.0001**	**2.65 (1.65** to **4.26)**	**.0001**
Previous treatment	**1.9 (1.2** to **3.1)**	**.0001**	**2.93 (1.4** to **6.1)**	**.0001**
Necrosis <90%	**1.8 (1.39** to **2.3)**	**.0001**	**1.63 (1.24** to **2.1)**	**.003**

NOTE. The bold values signify statistically significant variables (*P* < .05).

Abbreviations: HR, hazard ratio; LDH, lactate dehydrogenase; NVB, neurovascular bundle; SAP, serum alkaline phosphatase.

In the metastatic cohort, failure to complete treatment protocol, poor histological necrosis, tumors not amenable to local treatment, and >10 metastases were also predictive of superior EFS on multivariable analysis (Table 4).

**TABLE 4:** Metastatic Osteosarcoma: Factors Included in the Model for the Whole Cohort

Variables	HR	*P*	95% CI
Failure to complete protocol	5.29	.000	2.60 to 10.7
Necrosis <90%	1.96	.009	1.18 to 3.26
Not amenable to local treatment	1.35	.234	0.83 to 2.20
>10 metastases	2.44	.019	1.16 to 5.13

Abbreviation: HR, Hazard ratio.

#### ES

On multivariable analysis, failure to complete treatment protocol, previous treatment, residual viable tumor, and tumor size >8 cm were independent predictors for inferior EFS for nonmetastatic tumors (Table 5).

**TABLE 5:** Nonmetastatic Ewing's Sarcoma: Factors Included in the Model for the Whole Cohort

Variables	HR	*P*	95% CI
Failure to complete protocol	**2.84**	**.042**	1.03 to 7.80
Previously treated	**6.37**	**.003**	1.80 to 22.0
Necrosis <100%	**8.73**	**.002**	2.16 to 35.30
Tumor size >8 cm	**2.64**	**.019**	1.04 to 6.70

NOTE. The bold values signify statistically significant variables (*P* < .05).

Abbreviation: HR, Hazard ratio.

In the metastatic cohort, failure to complete protocol and tumors not amenable to local treatment independently predicted inferior EFS (Table 6).

**TABLE 6:** Metastatic Ewing's Sarcoma: Factors Included in the Model for the Whole Cohort

Variables	HR	*P*	95% CI
Failure to complete protocol	3.88	.002	1.63 to 9.23
Not amenable to local treatment	10.60	.019	1.46 to 77.8

Abbreviation: HR, Hazard ratio.

### Model

#### Nonmetastatic OGS

**FIG 2:**
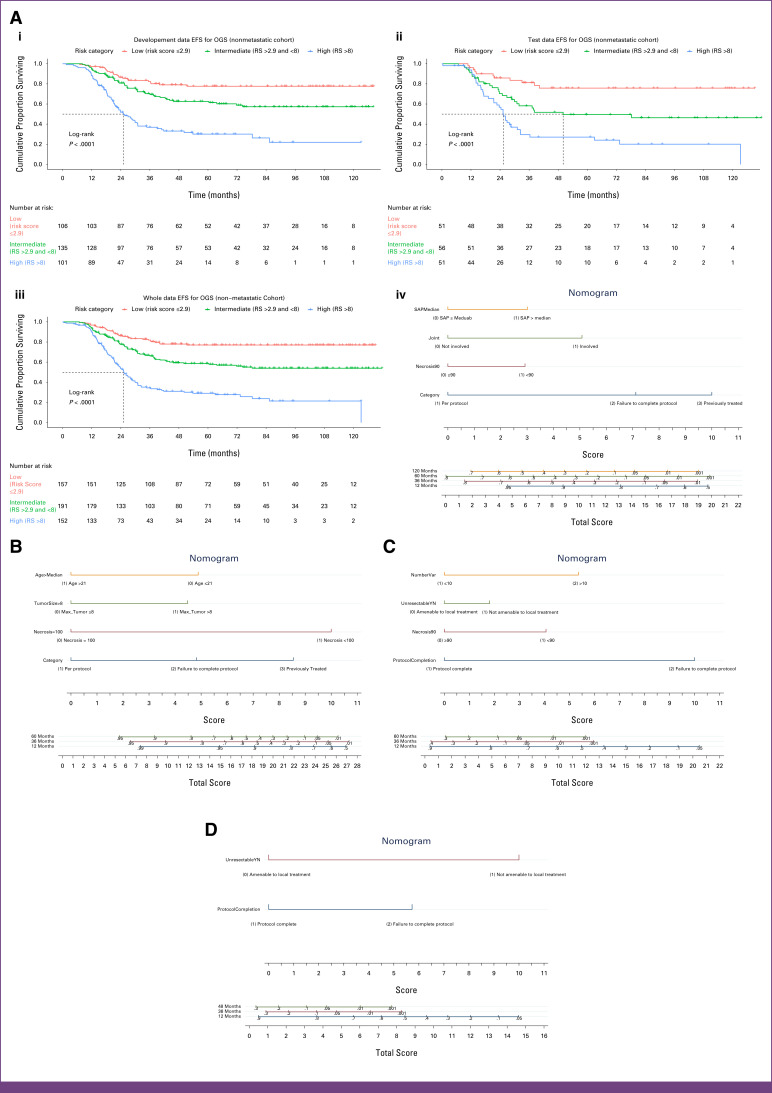
(A) [i] Kaplan-Meier curve depicting EFS for patients with nonmetastatic OGS: derivation cohort (n = 342). [ii] Kaplan-Meier curve depicting EFS for patients with nonmetastatic OGS: derivation cohort (n = 158). [iii] Kaplan-Meier curve depicting EFS for patients with nonmetastatic OGS: derivation cohort (n = 500). (B) Nomogram depicting the prognostic model for nonmetastatic ES. (C) Nomogram depicting the prognostic model for metastatic OGS. (D) Nomogram depicting the prognostic model for metastatic ES. EFS, event-free survival; ES, Ewing sarcoma: OGS, osteosarcoma; RS, risk score; SAP, serum alkaline phosphatase.

Variables contained in the model for the whole cohort included failure to complete protocol (8.7 points), previous treatment (10.0 points), necrosis <90%: 4.3 points, neurovascular bundle involvement: 4.4 points, joint involvement: 6.3 points, and SAP >median (204 U/L): 5.2 points (Table 7). In the validation cohort (n = 158), factors that emerged significant and are included in the final model were failure to complete protocol (7.1 points), previous treatment (10.0 points), necrosis <90%: 2.9 points, joint involvement: 5.1 points, and SAP >median (204 U/L): 3.0 points (Table 8). Neurovascular bundle involvement was excluded from the risk score model as it was not found to be significant in the validation cohort. Stratification was done by summation of the individual scores as follows: low risk (score <3), intermediate risk (3-8), and high risk (>8). Kaplan-Meier curves for EFS for the derivation (n = 342), validation (n = 158), and whole (n = 600) cohorts are depicted in Figure 2A[i], 2A[ii] and 2A[iii], with the nomogram depicted in Figure 2A[iv]. The c-index for whole, derivation, and validation data was 0.659, 0.664, and 0.651, respectively.

**TABLE 7:** Nonmetastatic Osteosarcoma: Factors Included in the Model for the Whole Cohort

	HR	*P*	95% CI
Failure to complete protocol	**2.66**	**.000**	1.65 to 4.26
Previously treated	**3.08**	**.000**	1.40 to 6.79
Necrosis <90%	**1.62**	**.001**	1.23 to 2.13
Neurovascular bundle involvement	**1.65**	**.006**	1.15 to 2.35
Joint involvement	**2.03**	**.000**	1.51 to 2.73
SAP >median (204 U/L)	**1.79**	**.000**	1.34 to 2.38

NOTE. The bold values signify statistically significant variables (*P* < .05).

Abbreviations: HR, hazard ratio; SAP, serum alkaline phosphatase.

**TABLE 8:** Nonmetastatic Osteosarcoma: Factors Included in the Model for the Validation Cohort

	HR	*P*	95% CI
Failure to complete protocol	**3.42**	**.000**	1.88 to 6.24
Previously treated	**5.64**	**.000**	2.24 to 14.19
Necrosis <90%	**1.65**	**.004**	1.17 to 2.33
Joint involvement	**2.40**	**.000**	1.71 to 3.37
SAP >median (204 U/L)	**1.68**	**.003**	1.19 to 2.36

NOTE. The bold values signify statistically significant variables (*P* < .05).

Abbreviations: HR, hazard ratio; SAP, serum alkaline phosphatase.

#### Nonmetastatic ES

Factors significant in the cohort and included in the final model were failure to complete protocol (4.8 points), previous treatment (8.5 points), necrosis <100% (10.0 points), and tumor size >8 cm (4.5 points), depicted in the nomogram (Fig 2B).

#### Metastatic OGS

Factors significant in the cohort and included in the final model were failure to complete protocol (10.0 points), not amenable to local treatment (1.8 points), necrosis <90% (4.1 points), and >10 metastases (5.4 points), depicted in the nomogram (Fig 2C).

#### Metastatic ES

Factors significant in the cohort and included the final model were failure to complete protocol (5.7 points) and not being amenable to local treatment (10 points), depicted in the nomogram (Fig 2D).

## DISCUSSION

In this study, we analyzed a prospectively maintained cohort of AYA with OGS treated at our center using a uniform non-HDMTX–based protocol OGS-12 and ES treated with our institutional standard EFT-2001 protocol. We formulated and validated prognostic scores separately for metastatic and nonmetastatic OGS and ES on the basis of baseline clinical factors and tailored to a unique population of patients treated in a resource-constrained setting with our in-house, low-cost, institutional standard protocols that have previously demonstrated that survival outcomes are comparable with those published from Western countries.^12-15^

For nonmetastatic AYA sarcomas, features of high tumor burden including higher baseline SAP, neurovascular bundle involvement, and joint involvement predicted poor EFS. This is consistent with published literature—SAP and LDH are surrogates of osteoblastic activity, and thus, elevated levels may indicate increased disease aggressiveness.^4^ Additionally, vascular involvement noted radiologically in pretreatment magnetic resonance imaging has been found independently to be a risk factor for OS and EFS in patients with Enneking IIB primary OGS involving extremities.^16^ Poor histological necrosis also predicted inferior EFS, which is well described in the existing literature.

Importantly, failure to complete treatment and previous treatment independently predicted inferior EFS. This is particularly significant in LMIC, with high treatment abandonment rates as well as failure to complete treatment protocol due to various issues including financial challenges, lack of education, and motivation for treatment and logistic issues relating to the need to travel long distances and stay far from the patient's hometown for the duration of treatment due to unavailability of cancer care near the place of residences. Thus, prompt referral to dedicated centers specializing in cancer care and awareness of primary health care physicians to recognize bone sarcomas and avoid inadvertent inappropriate medical or surgical intervention are of paramount importance.

For ES, tumor size >8 cm was an additional prognostic factor, consistent with the reported literature.^17,18^ In the metastatic cohort, failure to complete treatment protocol, poor histological necrosis, tumors not amenable to local treatment, and >10 metastases were also predictive of inferior EFS on multivariable analysis. This underscores the importance of multimodality clinics to identify tumors amenable to curative treatment and avoid overtreatment for patients with extensive disease. It has been previously observed that OGS presenting only with lung metastases has better survival outcomes than metastases at other sites.^19^

The factors that emerged prognostically significant for our patients were similar to those described in data from centers using HDMTX-based protocols, indicating the wide applicability of our results. For non-HDMTX regimens, particularly prevalent in LMIC, data on prognostic factors are scarce with only few observational studies.^20-22^ Histologic response to chemotherapy has been uniformly reported to be a predictive factor in these studies. A study from Brazil has additionally reported presence of metastases at baseline, primary tumor site, and type of surgery (amputation *v* limb sparing) as prognostically significant.^23^ Nevertheless, the lacunae for studies specifically targeting bone sarcomas in LMIC remains apparent. Smaller sample sizes of existing studies, observational nature, and nonuniformity of treatment protocols make generalizability of these results challenging. A potential strategy to overcome these shortcomings include collaborative efforts with multi-institution studies to enhance understanding of OGS in LMIC. Importantly, several existing studies have included patients with both nonmetastatic and metastatic tumors, with widely differing outcomes. We have addressed this limitation by separately analyzed homogeneously treated populations of metastatic and nonmetastatic OGS and ES.

In our study, we developed separate prognostic models for nonmetastatic and metastatic OGS and ES on the basis of the prognostic factors identified. We validated the risk score for nonmetastatic OGS and demonstrated effective discriminative ability for EFS between the three risk groups. For the other cohorts (metastatic OGS, and nonmetastatic and metastatic ES), we developed prognostic models depicted as nomograms, although validation could not be separately performed due to the limited sample size. Available prognostic models for risk stratification in OGS involve variable treatment modalities, clinician preferences, surgical expertise, and chemotherapy protocols that preclude generalizability of these models.^24-27^ For ES, existing models have largely used treatment-related factors such as choice of local treatment, factors that vary in different centers due to individual clinician preference, and expertise.^28-33^ In developing our prognostic models, we have analyzed homogeneously treated cohorts and reinforced the importance of baseline indicators of tumor aggressiveness, chemosensitivity as indicated by histologic response to chemotherapy, and general treatment-related factors unique to patients in LMIC settings, such as failure to complete treatment protocol and previous inadvertent treatment before referral to advanced oncology centers.

The prognostic models we report are important and unique as they are derived from a prospective analysis of homogeneously treated AYA in an LMIC setting. The specific challenges faced by this vulnerable subset of patients have been stressed upon. The wide implications of our results include health care policymaking, in the context of need for early identification by appropriate awareness and training of primary care physicians and timely referral to sarcoma reference centers, and the importance of strategies to improve protocol completion by efforts such as targeted nutritional intervention, extended growth factor support, and patient navigation facilities. Risk stratification of patients aids selection of patients who would benefit from aggressive treatment as well as those who may achieve comparable outcomes with less aggressive modalities with improved quality of life. Traditionally, escalation of treatment for patients with high-risk disease has been based on histologic response after completion of NACT.^34,35^ Use of other important prognostic factors as described in our study may be used to identify patients who may benefit from a less intensive approach such as metronomic chemotherapy regimens and early incorporation of palliative care, particularly in resource-constrained settings.^36,37^

The prognostic implication of common social challenges has been highlighted, including previous inadvertent treatment and the importance of compliance in the form of protocol completion. We did not analyze socioeconomic strata due to unavailability of uniform data; however, surrogate parameters including compliance as assessed by treatment duration and protocol completion, as well as previous inadvertent treatment were analyzed. Importantly, the majority of our patients are constrained financially and socially and depend on financial aid from various government schemes for cancer treatment. Another study from India has evaluated location of primary residence of the patient and the distance of the residence from the hospital and not found prognostic significance for OGS.^22^

This is the largest, single-center prospective analysis of prognostic factors for AYA with bone sarcomas globally, with the first prognostic model tailored for AYA with generalizable results for LMIC.

Limitations include the relatively shorter follow-up; however, the median follow-up was around 6 and 72 months, respectively, in OGS and ES, which is reasonable. Continued follow-up over an even longer time period of up to 10 years will further strengthen the value of these observations. There were lower number of AYA with ES, which is as expected for a rare cancer. Additionally, the prognostic scores for metastatic OGS and for both nonmetastatic and metastatic OGS could not be validated in a separate validation cohort due to lower sample sizes. Socioeconomic backgrounds of our patients were not assessed with objective scales such as the Kuppuswamy scale; however, surrogate parameters such as treatment duration as an indicator for compliance, previous inadvertent treatment, and protocol completion were included in the analysis.

We conclude that simple prognostic models predict the prognosis in individual homogeneous cohorts of AYA patients with nonmetastatic and metastatic OGS and ES. In addition to conventional prognosticators, failure to complete the treatment protocol (all cohorts) and the disease not being amenable to curative treatment (metastatic cohort) were associated with inferior outcomes. These simple prognostic models are effective and with wide applicability including in LMIC and merit appropriate recognition.
